# A Survey Study on the Current Veterinary Practice and Attitudes to Anaesthesia and Analgesia for Spay Surgery in the United Kingdom

**DOI:** 10.1002/vms3.70151

**Published:** 2025-01-06

**Authors:** Joanna Martino‐Boulton, Iliana Antonopoulou, Hannah Pinnock, Chiara Adami

**Affiliations:** ^1^ Department of Veterinary Medicine University of Cambridge Cambridge UK

**Keywords:** canine pain, feline pain, neutering, nociception, spay surgery

## Abstract

**Background:**

In the United Kingdom, spay surgery is routinely performed in dogs and cats by general practitioners. Data from a decade ago showed that, despite an increased attentiveness of veterinarians to peri‐operative pain compared to the past, analgesia could be further improved.

**Objectives:**

To investigate the current veterinary practice and attitude towards anaesthesia and analgesia for spay surgery in the United Kingdom.

**Methods:**

An electronic questionnaire composed of 57 questions organised in 6 sections was designed using the Checklist for Reporting Results of Internet E‐Surveys (CHERRIES guidelines) and distributed online via a hyperlink. Participants were recruited through both personalised email invitation and publication of the hyperlink on social media. Data were analysed with descriptive statistics, analysis of means and analysis of proportions, using commercially available software.

**Results:**

Entries from 150 participants were used for data analysis. The proportion of participants who were confident in treating pain did differ by decade of graduation, with a lower proportion of confident colleagues graduated before 2001 (6%) and from 2021 (14%), compared to those graduated in the decades 2001–2010 (43%) and 2011–2020 (37%) (*p* = 0.007). Colleagues reported to implement multimodal analgesia for spay procedures of cats and dogs in 43% and 44% of cases, respectively. The proportions of participants who reportedly used locoregional blocks, mostly with lidocaine, in dogs (82%), were higher than that in cats (43%) (*p* < 0.001). Post‐spay surgery pain was perceived by the participants as more intense in dogs than in cats (*p* < 0.001).

**Conclusions:**

Despite an overall good level of attentiveness of British veterinary professionals to feline and canine analgesia during and following spay surgery, this study identified as areas of improvements perception and assessment of feline pain and implementation of locoregional anaesthetic techniques, particularly in cats.

## Graphical abstract

• There was an overall good level of attentiveness of British veterinary professionals to feline and canine analgesia during and following spay surgery.

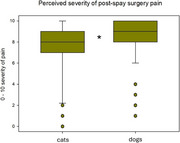

## 1 Introduction

A recent survey study reported that 75% of veterinarians practicing in the United Kingdom recommend neutering of non‐breeding dogs, which has been estimated that approximately 50% and 90% of dogs and cats, respectively, are neutered in this country (Cosgrove 2024). Surgical neutering is an elective procedure most commonly performed, in juvenile companion animals, by general veterinary practitioners. Although male castration is often perceived as relatively easy to perform, spay surgery involves a laparotomy, which makes this procedure technically more challenging and usually of longer duration.

Anaesthesia and analgesia of small animal species have evolved exponentially during the last two decades, with the introduction of a variety of analgesic molecules now commercially available and licensed for dogs and cats, as well as with the increasingly common use of locoregional analgesic techniques such as incisional infiltration and transversus abdominis block (Cavaco et al. 2022; Garbin et al. 2022; Fudge et al. 2020; Herlofson et al. 2023; Kazmir‐Lysak et al. 2023). A study conducted in 2015 showed an improvement of the attitude of veterinarians towards attentiveness to peri‐operative pain, as demonstrated by the increased tendency to prescribe both peri‐operative non‐steroidal anti‐inflammatory drugs (NSAIDs) and multimodal analgesia compared to the past (Capner, Lascelles, and Waterman‐Pearson 1999, Hunt et al. 2015). However, this published research highlighted that the use of local anaesthetics was uncommon and peri‐operative pain was perceived as more intense in cats than in dogs, suggesting that animal welfare and current practice could be further improved (Hunt et al. 2015). Other studies investigated the attitude of Spanish and Swiss veterinarians towards animal pain and concluded that analgesia was adequately addressed, although these reports did not specifically look at elective spay surgery (Perret‐Gentil et al. 2014; Menéndez, Cabezas, and Gomez de Segura 2023). On the contrary, one study on the opinion and knowledge of Italian veterinarians on abdominal visceral pain reported a disappointingly low proportion of participants using pain scoring systems (Catanzaro et al. 2016).

The aim of this survey study was to investigate the current veterinary practice and attitude towards anaesthesia and analgesia for spay surgery in the United Kingdom.

It was hypothesised that registered veterinary nurses (RVN) and general practitioners in the United Kingdom would conform to high professional standards, characterised by the use of both modern anaesthetic protocols, inclusive of drugs such as alfaxalone, pure‐μ opioids and novel NSAIDs that have been introduced in the veterinary market during the last decade, and advanced and validated pain scales to assess and recognise post‐spay pain in cats and dogs.

## 2 Materials and Methods

### 2.1 Study Design

This prospective, observational e‐survey study was based on a questionnaire delivered through a commercially available software (Qualtrics XM Institute 2019, UT, USA) and accessed online via a hyperlink. Both the study and the questionnaire were designed using the Checklist for Reporting Results of Internet E‐Surveys (CHERRIES guidelines) as guidance (Eysenbach 2004; Shah et al. 2006).

Prior to accessing the questionnaire, participants were requested to tick a box to grant their consent for the use of data for publication.

Duplicate entries from the same users were prevented by both the use of cookies and identification and recording of the internet protocol (IP) addresses of each participant's computer. The participants’ email addresses were requested during registration to the survey for the purpose of assigning to each subject a unique user identifying code; however, they were not disclosed to the researchers or linked to the responses to ensure anonymity. All responses were completely anonymised, and neither the personal details of the participants nor their IP addresses, recorded by the system to prevent duplication, were displayed. Access to electronic data gathered from the study was password‐protected, with the password known only the primary investigators. Data were initially stored on Qualtrics and then, at the end of data collection, transferred on a Microsoft Excel working file for subsequent statistical analysis.

### 2.2 Recruitment of Participants

Potential participants to the survey study were identified as all the veterinarians and RVN providing anaesthesia for elective feline and canine spay surgery in the United Kingdom. Colleagues known by personal acquaintance by the authors who were identified as eligible to complete the survey were contacted directly via email, and a personalised invitation to complete the survey was sent to them. The hyperlink to complete the survey was circulated by VetPartners to their small animal practices in the United Kingdom and shared on social media via publication on the Facebook pages of the groups ‘Vet mums’ and ‘Vetvoices’. Additionally, a number of practices were randomly selected by searching their contact details on the Royal College of Veterinary Surgeons (RCVS 2024) website by location.

### 2.3 Questionnaire

The questionnaire was composed of a total number of questions equal to 52, organised into 6 separate sections, entitled Part 1 (demographics and general data, 5 questions), Part 2 (feline spay anaesthesia and analgesia protocols, 15 questions), Part 3 (Feline pain assessment after spay surgery, 4 questions), Part 4 (Canine spay anaesthesia and analgesia protocols, 18 questions), Part 5 (Canine pain assessment after spay surgery, 5 questions) and Part 6 (confidence in assessing and treating pain and attitude towards pain management, 5 questions). All questions but one (year of graduation, Part 1) were multiple choice questions with one single best answer. Response templates for continuous variables were in form of incremental numerical ranges (e.g., 0–12, 13–24 h), whereas categorical variables were based on both frequency and level of agreement Likert scale, as per CHERRIES guidelines.

When the questionnaire was delivered online, the order of questions within each part, as well as the order of Part 2–3 (feline) and 4–5 (canine), was randomised to prevent question order bias.

In the questionnaire, ‘peri‐operative’ was defined as the time elapsing from premedication to 6 h after surgery, whereas multimodal analgesia was defined as the combination of different classes of systemic analgesics, with or without the complementary use of locoregional anaesthesia techniques.

### 2.4 Data Analysis

The completion rate was calculated by dividing the number of participants who successfully completed the questionnaire by the total number who accessed the questionnaire but failed to submit a complete set of responses. Entries from participants who completed less than 60% of the questionnaire were excluded from data analysis.

Descriptive statistics, analysis of means and analysis of proportions were used. The Kolmogorov–Smirnov and Shapiro–Wilk tests were used to assess data distribution. The Chi‐square test of independence was used in the analysis of contingency tables to determine whether paired of categorical variables were independent. For this purpose, the variable ‘year of graduation’ was categorised into four subgroups: A (before 2001), B (2001–2010), C (2011–2020) and D (from 2021). Either one‐way analysis of variance or Kruskal Wallis analysis of variance on ranks, depending on data distribution, was used to compare, respectively, the means and medians of continuous variables (e.g., expected level of pain based on a 0–10 scale) of three or more independent groups (e.g., participants grouped on the basis of year of graduation). Either the *t*‐test or the non‐parametric Mann–Whitney *U*‐test was used to compare continuous variables between feline and canine. Cronbach's alpha test was used to measure internal consistency of the questionnaire items. The Fisher exact test was used to compare the proportion of participants reportedly using specific agents/techniques in dogs and cats.

Commercially available statistical software was used (SigmaStat 3.5 and SigmaPlot 10, Systat; SPSS, version 28, IMB Corp.). *p* values of 0.05 or less were considered statistically significant.

## 3 Results

Descriptive data were reported as numbers of participants and proportions. For data obtained from questions secondary to a primary inquiry, proportions were calculated on the number of subjects who answered the primary enquiry.

A total of 153 participants initially accessed the survey, of which only 129 completed the survey, accounting for a completion rate of 0.84. Data from three participants who completed less than 60% of the questionnaire were excluded from the study, leaving a total of 150 entries used for data analysis.

Cronbach's alpha coefficient for internal consistency of the questionnaire was 0.61 (Cronbach's *Q* between 56 items: 1287, with 22 degrees of freedom and *p* < 0.001).

In the results reported below, when the sum of the proportions for a specific set of data does not reach 100%, the absent proportion represents participants who did not answer that specific question. Proportions were calculated accounting for the non‐respondents.

### 3.1 Demographics and General Data

Most of the participants were females (125, 82%) and only 22 were males (2%). Of the 53 participants who answered the question on their qualification, 38 (25%) were members of the RCVS (MRCVS) and 15 (10%) were RVN. The most represented decades of graduation were 2001–2010 (40, 27%) and 2011–2020 (42, 28%), with only 12 (8%) and 9 (6%) participants having graduated before 2001 and after 2021, respectively. Most participants (128, 85%) worked in small animal general practice, 17 of them (11%) in mixed general practice and only 5 (4%) in referral hospitals. The most common location was towns (98, 65%), followed by cities (27, 18%) and rural areas (25, 17%). For most participants (119, 79%), 91%–100% of the working hours were allocated to small animal practice, whereas for 11 (7%) and 10 (7%) of them, this only accounted for 71%–90% and 51%–70%, respectively. For six participants, small animal allocation time was only 31%–50%.

### 3.2 Confidence in Assessing and Treating Pain and Attitude Towards Pain Management

Most participants reported to feel confident in recognising (124, 83%) and treating (137, 92%) pain in cats and dogs after spay surgery, with only 21(14%) and 8 (5%) of them feeling somewhat confident. For most (60, 40%), the preferred source of information on feline and canine pain assessment was continuing professional development, followed by scientific journals (23, 15%) and university lectures (2, 1%). The majority of participants (62, 41%) strongly agreed that veterinary nurses should be primarily responsible for assessing postoperative pain in dogs and cats, whereas only 27 participants (18%) strongly agreed that this should fall within the competencies of veterinarians.

There was no association between the level of confidence of the participants in recognising and assessing pain and their decade of graduation (*p* = 0.089). However, the proportion of participants who were confident in treating pain did differ by a decade of graduation, with a lower proportion of confident colleagues graduated before 2001 (6%) and from 2021 (14%), compared to those graduated 2001–2010 (43%) and 2011–2020 (37%) (*p* = 0.007). Similarly, the proportion of participants confident in using pain scales was higher between the 2001–2010 (40%) and 2011–2020 (38%) graduates, compared with the oldest (11%) and youngest (11%) generations (*p* = 0.037). There was no association between the participants’ decade of graduation and the frequency of use of both the pain scales and multimodal analgesia in dogs and cats (*p* = 0.086).

**FIGURE 1:**
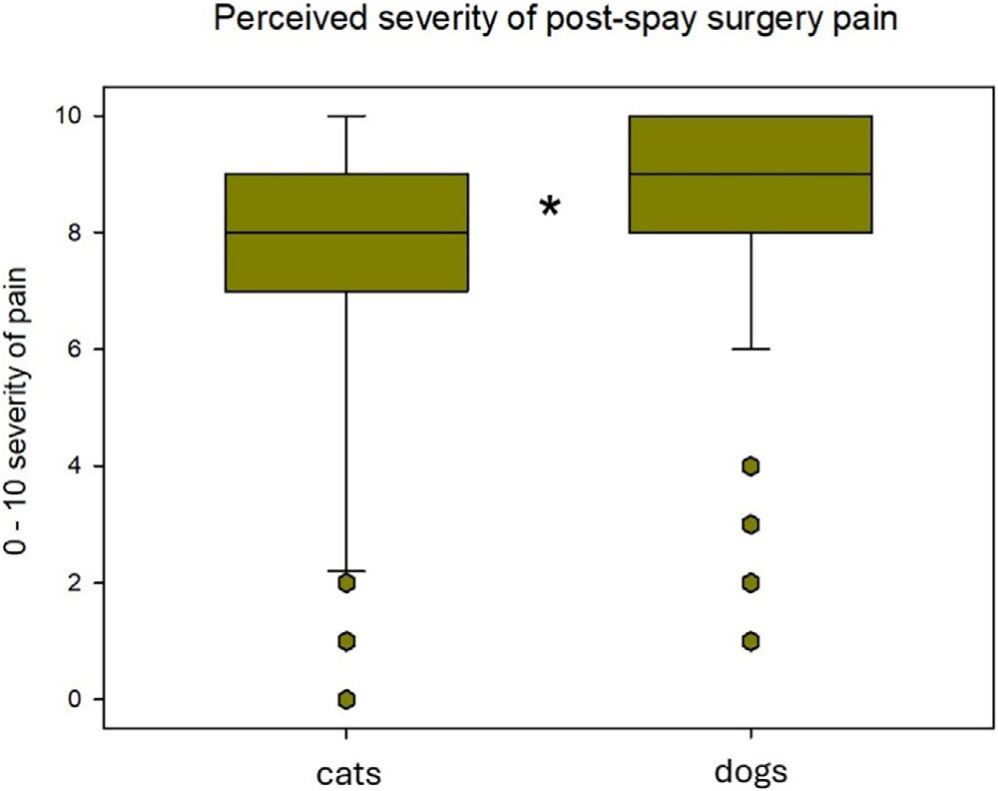
Perceived postoperative pain in cats and dogs after spay surgery. Box plots representing the perceived severity of feline and canine pain in the post‐spay surgery period (extending from tracheal extubation to 24 h after surgery) in case no analgesics were administered, on a 1–10 scale, by 150 survey study participants. The boxes represent the second and third quartiles, with the medians being represented by the horizontal line inside them. The vertical lines either side of each box represent the lower (25%) and upper (74%) quartiles; the dots represent the outliers. *: Statistically significant (*p* < 0.001).

The proportion of participants who strongly felt that nurses should be primarily responsible for pain assessment did differ by year of graduation, with 100% of the graduates from 2021, 58% of the graduates from 2011–2020, 38% of the graduates from 2001–2010 and 37% of the graduates before 2001 (*p* = 0.014). Post‐spay surgery pain was perceived by the participants as more intense in dogs than in cats (Figure 1; *p* < 0.001).

### 3.3 Feline Spay Anaesthesia and Analgesia Protocols

**FIGURE 2:**
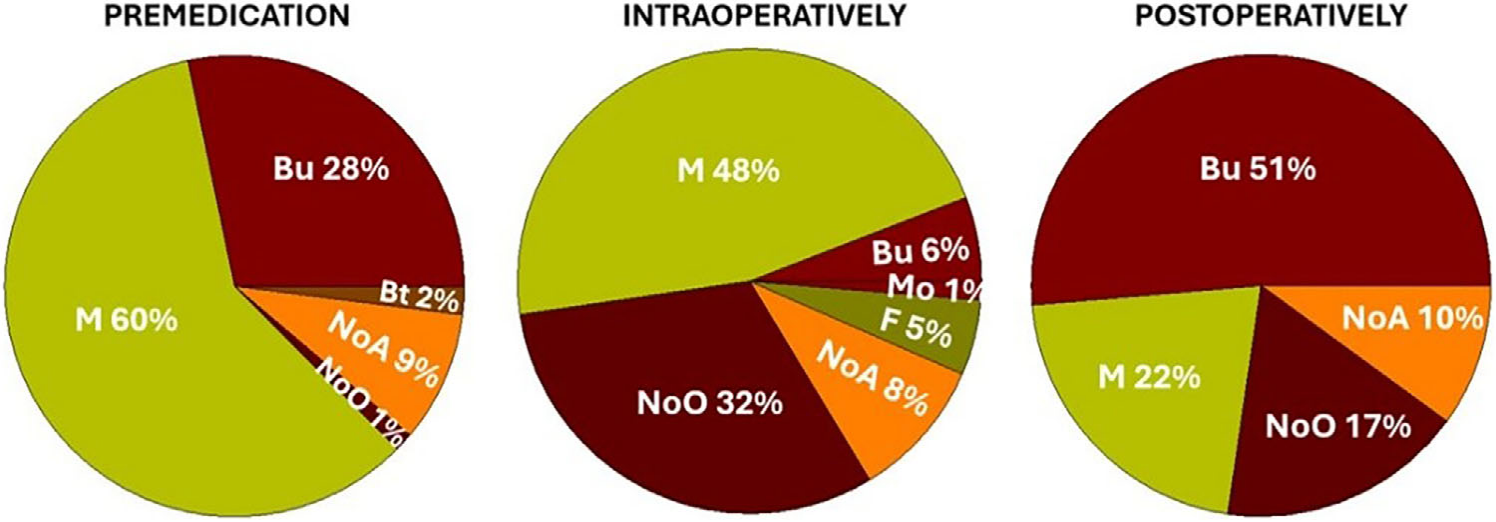
Peri‐operative use of opioids in cats undergoing spay surgery in the United Kingdom. Proportions are calculated on a total of 150 participants. Those who reportedly used intraoperative buprenorphine either had used it also for premedication or had not implemented premedication with any opioid. Bt, butorphanol; Bu, buprenorphine; F, fentanyl; M, methadone; Mo, morphine; NoA, no answer to the question; NoO, no opioids given.

The frequency of use of an alpha 2 adrenoreceptor agonist as part of premedication was ‘always’ in 77 cases (51%) followed by ‘most of the times’ (52, 34%) and ‘sometimes’ (7, 5%), whereas the preferred agent for anaesthetic induction was propofol (63, 42%), followed by alfaxalone (23, 15%) and a propofol/ketamine combination (7, 5%). A total of 82 (54%) participants reported to consistently use inhalational anaesthesia as part of their protocol, whereas only 2 (1%) never used it and the remaining 52 (35%) used it but not every time. Data pertaining to peri‐operative use of opioids are presented in Figure 2.

The frequency of implementation of multimodal analgesia ranged from ‘always’ (109, 73%), to ‘most of the times’ (21, 14%) and ‘sometimes’ (8, 5%). A total of 59 participants (39%) reported to never use locoregional techniques (both splash blocks and infiltrations); for the 64 (43%) who reportedly used them, the preferred local anaesthetic was lidocaine (43, 67%), followed by bupivacaine (21, 33%).

When asked about what they believed the duration of postoperative pain would be, 51 participants (34%) responded 2–4 days, 45 (30%) 5–7 days, 16 (11%) 8–10 days, 12 (8%) up to 14 days and 7 (5%) less than a day. A total of 124 participants (83%) reportedly used pain scales in the postoperative period, the preferred scale being the Glasgow Feline Composite Measure Pain Scale (CMPS—Feline; Reid et al. 2017) (103, 83%), followed by the Feline Grimace Scale (Evangelista et al. 2019) (21, 17%).

### 3.4 Canine Spay Anaesthesia and Analgesia Protocols

**FIGURE 3:**
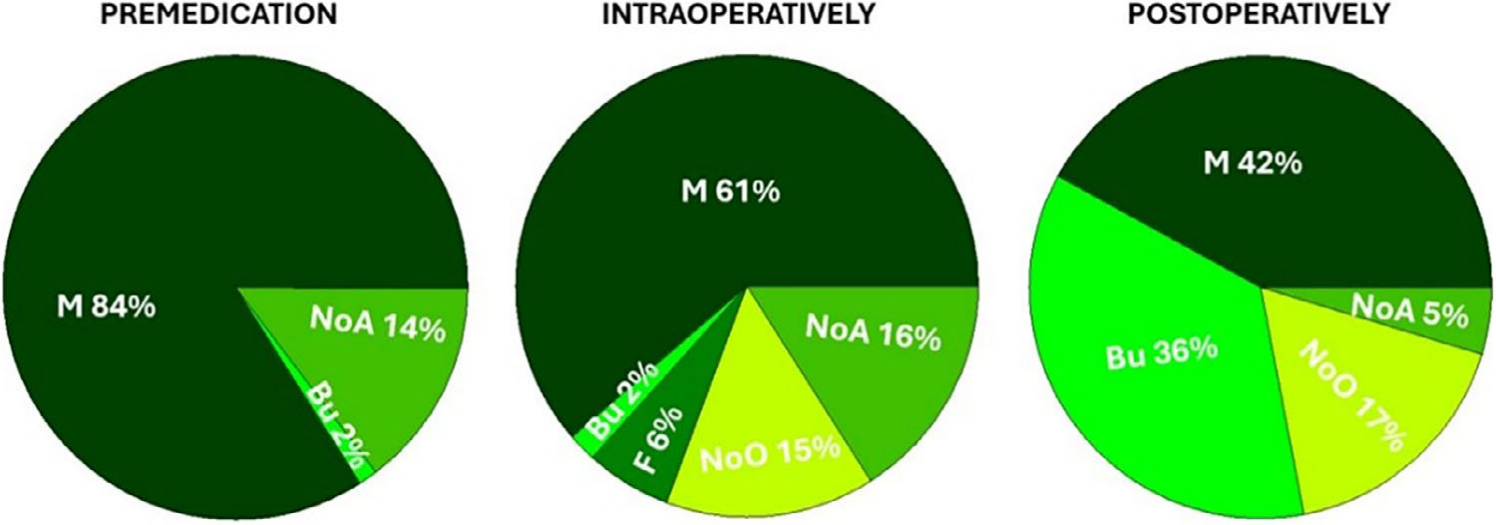
Peri‐operative use of opioids in dogs undergoing spay surgery in the United Kingdom. Proportions are calculated on a total of 150 participants. Bu, buprenorphine; F, fentanyl; M, methadone; NoA, no answer to the question; NoO, no opioids given.

A total of 41 (28%) participants reportedly avoided the use of an alpha 2 adrenoreceptor agonist as part of premedication; for those who used them, the frequency of use of was ‘always’ in 27 cases (17%) followed by ‘sometimes’ (26, 17%), ‘most of the times’ (20, 13%) and half of the times (14, 10%). The preferred agent for anaesthetic induction was propofol (100, 67%), followed by alfaxalone (20, 13%) and a propofol/ketamine combination (6, 4%). A total of 125 (84%) participants reported to always use inhalational anaesthesia as part of the protocol, whereas only 3 of them (1%) used it but not every time. Data pertaining to peri‐operative use of opioids are presented in Figure 3. Participants reportedly used buprenorphine in premedication for both species; however, the proportion was higher for cats (42, 28%) than for dogs (3, 2%) (*p* < 0.001).

The frequency of implementation of multimodal analgesia ranged from ‘always’ (111, 74%), to ‘most of the times’ (11, 8%), ‘half of the times’ (2, 1%) and ‘sometimes’ (8, 5%). Only 3 participants (1%) reported to never use locoregional techniques (both splash blocks and infiltrations); of the 123 (82%) who reportedly used them, only 85 (69%) expressed their preferences, namely, lidocaine (56, 66%) and bupivacaine (29, 34%). The proportions of participants who reportedly used locoregional blocks, mostly with lidocaine, in dogs (82%), were higher than that in cats (43%) (*p* < 0.001).

Data pertaining to the peri‐operative use of NSAIDs in both dogs and cats are reported in Table 1.

**TABLE 1:** Data pertaining to perioperative use of non‐steroidal anti‐inflammatory drugs (NSAIDs) for feline and canine spay surgery in the United Kingdom obtained from 150 participants to a survey study.

	Feline spay *n* (%)	Canine spay *n* (%)
Peri‐operative use of NSAIDs	124 (83)	77(51)
No perioperative use of NSAIDS	9 (6)	0 (0)
Did not answer	17 (11)	73 (49)

NSAIDs preference		
Meloxicam	72 (48)	76 (51)
Robenacoxib	2 (1)	0 (0)
Carprofen	0 (0)	22 (15)
Deracoxib	0 (0)	2 (1)
Did not answer	76 (51)	50 (33)

Duration of postoperative treatment		
Up to 24 h	0 (0)	0 (0)
2–5 days	102 (68)	72 (48)
>5 days	22 (15)	28 (19)
Did not answer	26 (17)	50 (33)

Paracetamol was reportedly added to the peri‐operative analgesic plan by 115 participants (77%), but only 100 (89%) participants using paracetamol reported the duration of the treatment (Table 1). Regarding drug combinations, 43 (28%) participants reportedly combined NSAIDs and paracetamol, whereas 73 (49%) preferred to use the NSAID alone.

When asked about what they believed the duration of postoperative pain would be, 51 participants (34%) responded 5–7 days, 41 (28%) 2–4 days, 19 (12%) 8–10 days, 11 (7%) up to 14 days and 3 (2%) less than a day. A total of 138 participants (92%) answered the question pertaining to pain scales, of which 127 (92%) reportedly used pain scales in the postoperative period, whereas the remaining 11 (8%) did not; for those who used them, the preferred scale was the Glasgow CMPS (Holton et al. 2001) (113, 89%), followed by the CMPS—short form (Reid et al. 2007) (12, 9%) and the Colorado State University Acute Pain Scale Canine (n.d.) (2, 2%).

## 4 Discussion

This study shows that veterinary professionals practicing in the United Kingdom use updated anaesthetic protocols, make efforts to detect postoperative pain, as demonstrated by the common use of validated pain scales, and use multimodal analgesia, when performing routine spay surgery in both dogs and cats. Nevertheless, the study findings also suggest that feline pain may still be partially neglected and addressed less attentively than in dogs.

Feline and canine peri‐operative nociception and pain were perceived differently, despite the surgical procedures being similar, with dogs perceived by the participants as prone to experience more intense nociception and pain than cats. This seems to be in‐line with the findings of a survey study conducted in 2015, which indicated that neutering was considered more painful in dogs than in cats (Hunt et al. 2015). One possible explanation to this finding is that flank laparotomy, the approach typically used for feline spay in the United Kingdom, may be perceived as less invasive and technically challenging than ventral laparotomy, which is most commonly performed in dogs (Burrow et al. 2006; Fuertes‐Recuero et al. 2024; Munif et al. 2022; Gauthier et al. 2015). Nevertheless, because the procedure is similar in dogs and cats and implies, in both species, isolation and traction of the ovarian pedicle and vascular ligation, it is reasonable to assume that the degree of visceral nociception would be similar in dogs and cats (Holzer‐Petsche and Brodacz 1999). Another possible explanation may be the stoic nature of cats, which are naturally more reluctant to show pain than dogs. Feline pain assessment is regarded as more challenging than in dogs even when performed by experienced veterinary personnel, and it is possible that cats may be neglected with this respect and their degree of pain underestimated (Steagall 2020).

The perception that pain associated to spay surgery would be less intense in cats compared to dogs may also explain the different attitude in selecting the opioid to be given in premedication, with a larger proportion of participants choosing buprenorphine over methadone for cats compared to dogs.

The use of locoregional anaesthetic techniques also differed between dogs and cats. In comparison with the findings of a study conducted in 2015, which reported that the majority of respondents did not use local anaesthetics techniques routinely for neither of the species, the current study showed that this attitude has improved towards canine patients, with 82% of respondents reportedly using both infiltrations and splash blocks; however, the participants using these techniques in cats were only 42% (Hunt et al. 2015). As a possible interpretation to this finding, there could be a cost element contributing to this discrepancy. Cat spays are generally priced relatively low in the United Kingdom, partly to encourage and promote population control; as a result, the addition of a lidocaine vial is proportionally a much larger increase in cost compared to bitch spay.

Regarding both the use of pain scales and treatment of peri‐operative pain, this study highlighted a generational effect, with both the youngest and oldest generations being less confident in using pain scales and treating pain compared to participants graduated between the decades 2001–2020. The decreased confidence of the older graduates may express the relatively recent implementation of the veterinary study curriculum with topics such as pain assessment and treatment, which in the past were possibly neglected and not specifically taught and addressed during the veterinary studies. The lower confidence expressed by the youngest generations of vets compared to the 2001–2020 graduates may be explained with their developing clinical maturity and inexperience. Interestingly, although the youngest and oldest generations felt least confident in using pain scales, this did not seem to affect their perceived ability and confidence to recognise pain in animals. This could reflect a tendency to rely their assessment on subjective evaluation rather than on specific and validated tools; however, how the participants assessed pain other than with the use of validated pain scales was not specifically asked to the participants during this survey.

Although the attitude towards pain assessment and management was generally good, it was reasonable to assume that attentiveness to analgesia—and, possibly, the degree of confidence in using validated pain scales—might differ between general practitioners and anaesthesia specialists, who become familiar with these tools during their training programme. Unfortunately, this hypothesis could not be tested owing to the too low number of participants who worked in referral centres. This was not an unexpected finding, as in the UK elective neutering of conventional small animal species is most performed by general practitioners rather than veterinary specialists.

This study has several limitations, the most important of which being the small size of the sample population, which may not be representative of the whole. On the basis of the RCVS (2024) website, at date there are more than 2700 RCVS‐accredited veterinary practices in the United Kingdom; as a result, the sample population of this study accounts for less than 6% of the whole population (RCVS 2024; website). Additionally, it is possible that, among the colleagues who had exposure to the link to access the survey, those who took the initiative to participate were naturally more receptive to animal pain and interested in anaesthesia and analgesia, causing a potential selection bias.

This study could not have been designed as a closed survey, a feature which is essential to calculate the participation rate and possibly better evaluate the representativeness and quality of the sample population. The large number of existing veterinary practices in the United Kingdom implied that personalised invitations to all the eligible participants would have been unrealistic.

Another limitation is that, although the questionnaire was designed in‐line with the CHERRIES guidelines and every effort was made to obtain good quality data, Cronbach's alpha coefficient was only 0.61, suggesting that the internal consistency of the questionnaire was questionable (Tavakol and Dennick 2011). This may potentially indicate that the questions were poorly worded and unable to reliably measure the outcome variables of interest; however, the lack of homogeneity in the responses may also reflect the diversity between practices and participants with respect to protocols and approaches.

In conclusion, this study shows that, although the attitude of British veterinarians and veterinary nurses towards peri‐operative analgesia for spay surgery is generally good, potential areas of improvements are perception, assessment and treatment of feline pain, as well as implementation of locoregional anaesthetic techniques, particularly in cats.

## Author Contributions

**Joanna Martino‐Boulton**: conceptualisation, investigation, data collection, project administration, writing–original draft. **Iliana Antonopoulou**: conceptualisation, writing–review and editing. **Chiara Adami**: conceptualisation, methodology, project administration, writing–original draft, writing–review and editing, data analysis. **Hannah Pinnock**: conceptualisation, data collection, writing–review and editing.

## Ethics Statement

This study was conducted under approval of the Ethics & Welfare Committee of the Department of Veterinary Medicine of the University of Cambridge (license number: CR778; approval date: 23 January 2024).

## Conflicts of Interest

The authors declare no conflicts of interest.

### Peer Review

The peer review history for this article is available at https://publons.com/publon/10.1002/vms3.70151.

## Data Availability

The data that support the findings of this study are all presented in the result section. Raw data may be made available by the corresponding author upon reasonable request.
